# Supplementary figures and images for: Evaluation of different culture media to support in vitro growth and biofilm formation of bacterial vaginosis-associated anaerobes
Source: PeerJ. 2020 Sep 10;8:e9917. doi: 10.7717/peerj.9917 (PMC7487148; doi:10.7717/peerj.9917)

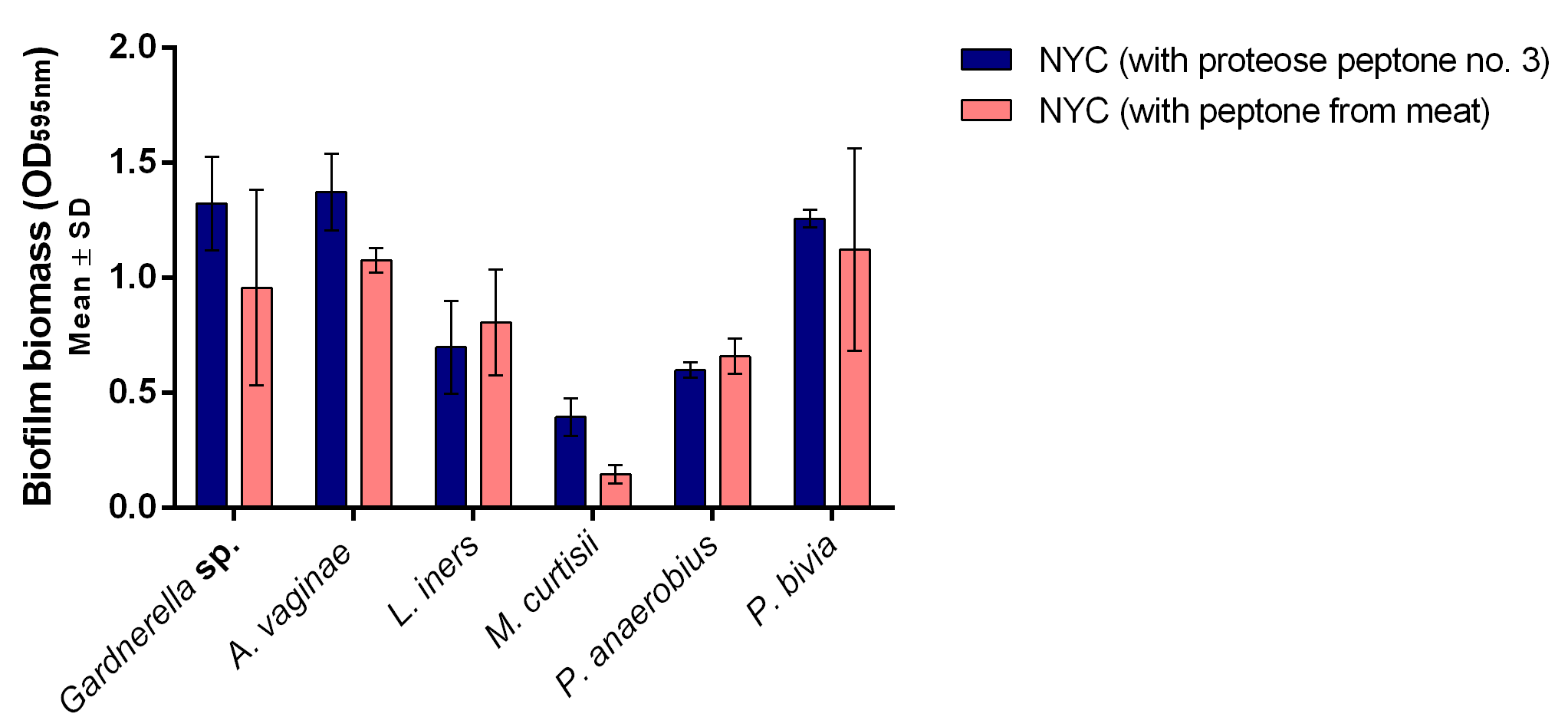

Supplement: Supplemental Information 1 — Biofilm biomass was quantified using the crystal violet staining assay. Results represent the average ± the standard deviation of three independent experiments performed with eight technical replicates. Statistical analysis was performed using two-way ANOVA and Sidak’s multiple comparisons test. No significant difference was found between biofilm-formation ability of BV-associated bacteria in the two tested culture media. [file peerj-08-9917-s001.png]

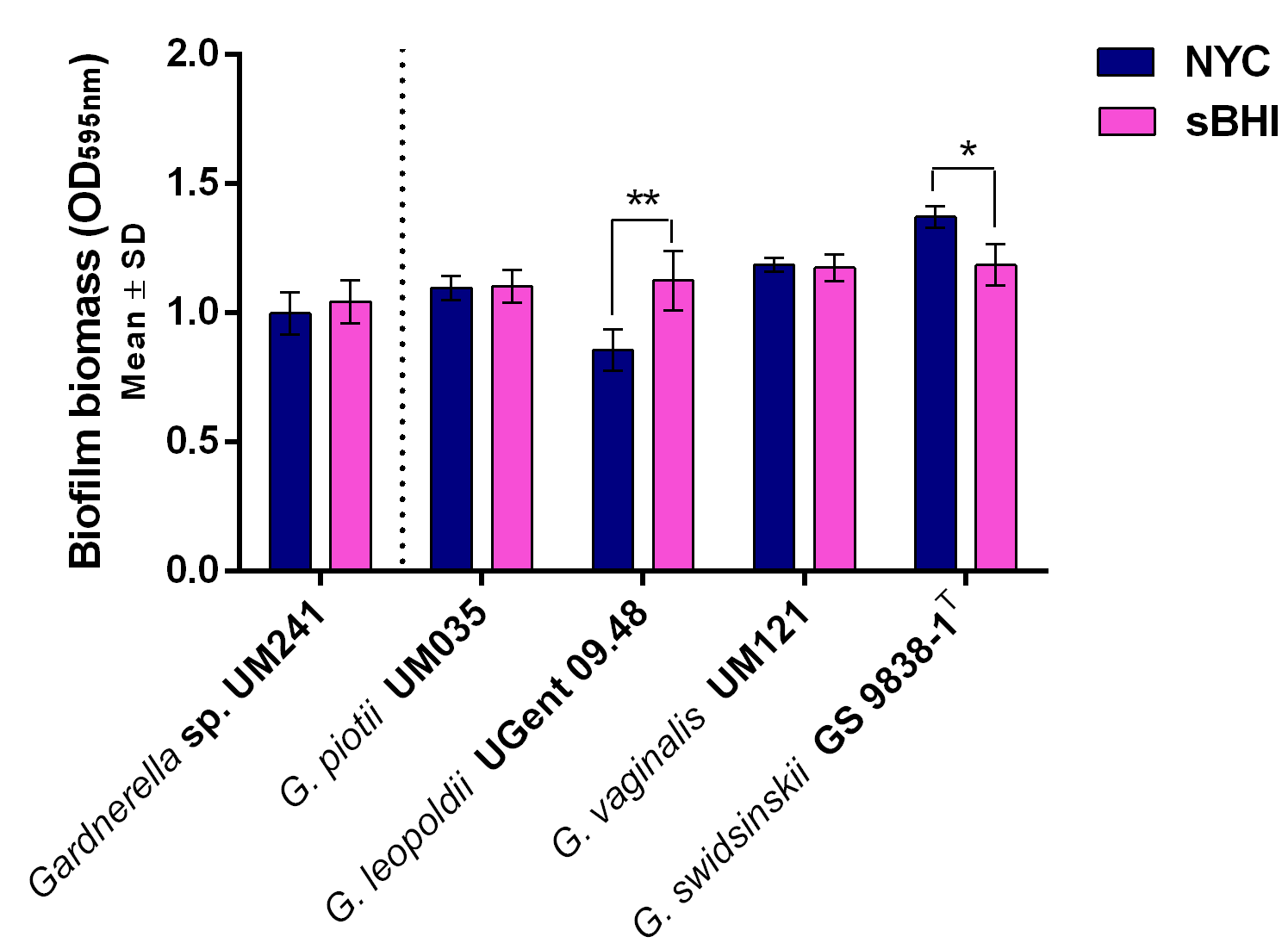

Supplement: Supplemental Information 2 — Biofilm biomass was quantified using the crystal violet staining method. Results represent the average ± the standard deviation of three independent experiments performed with eight technical replicates. Statistical analysis was performed using two-way ANOVA and Sidak’s multiple comparisons test. Significant differences between biofilm biomass formed in NYC and sBHI are represented with *p < 0.05 and **p < 0.01. [file peerj-08-9917-s002.png]
