# Supplementary material for: Evaluation of different culture media to support in vitro growth and biofilm formation of bacterial vaginosis-associated anaerobes
Source: PeerJ. 2020 Sep 10;8:e9917. doi: 10.7717/peerj.9917 (PMC7487148; doi:10.7717/peerj.9917)
Supplement: Supplemental Information 3 — 1 Statistical differences between bacterial planktonic growths in different culture media were analyzed with one-way ANOVA and Tukey’s multiple comparisons test, p < 0.05. a Statistical significance when comparing with BHV, b when comparing with BHV.Aa, c when comparing with NYC, d when comparing with NYC.Aa, e when comparing with SB, f when comparing with SB.Aa, g when comparing with sBHI, h when comparing with sBHI.Aa. 2 The effect of L-ascorbic acid on bacterial growth is presented as fold change relative to the growth in the medium without L-ascorbic acid (fold change = 1, control). This effect was classified as inhibitory (cut-off < 0.75 – fold change), neutral (0.75 ≤ fold change < 1.25), and stimulatory (cut-off ≥ 1.25 – fold change). [file peerj-08-9917-s003.docx]

**Supplemental Table S1.**

**Fold change in planktonic growth of BV-associated bacteria in the nine different culture media relative to OD_620nm_ values measured at T0h.**

| **Bacteria** | **Culture medium** | | | | | | | | |
| --- | --- | --- | --- | --- | --- | --- | --- | --- | --- |
|  | **BHV** | **BHV.Aa^2^** | **NYC** | **NYC.Aa^2^** | **SB** | **SB.Aa^2^** | **sBHI** | **sBHI.Aa^2^** | **mGTS** |
| ***Gardnerella* sp.**  Growth ratio, Mean±SD  *p^1^* | 1.1±0.1 | 1.1±0.1 | 9.8±2.1  a,b | 7.6±0.6  a,b | 0.9±0.2  c,d | 1.2±0.2  c,d | 1.1±0.3  c,d | 2.1±0.6  c,d | 1.6±0.1  c,d |
| ***Atopobium vaginae***  Growth ratio, Mean±SD  *p* | 0.8±0.2 | 1.1±0.1 | 10.4±2.1  a,b | 8.8±0.8  a,b | 1.0±0.1  c,d | 1.0±0.1  c,d | 1.0±0.1  c,d | 1.0±0.3  c,d | 1.2±0.1  c,d |
| ***Lactobacillus iners***  Growth ratio, Mean±SD  *p* | 1.2±0.2 | 1.2±0.3 | 9.9±1.9  a,b | 10.5±0.7  a,b | 0.8±0.1  c,d | 0.8±0.2  c,d | 0.9±0.1  c,d | 1.3±0.1  c,d | 1.2±0.1  c,d |
| ***Mobiluncus curtisii***  Growth ratio, Mean±SD  *p* | 1.1±0.1 | 1.0±0.3 | 1.4±0.1 | 1.4±0.3 | 1.2±0.1 | 1.4±0.1 | 1.2±0.1 | 1.6±0.5 | 0.9±0.1  h |
| ***Peptostreptococcus anaerobius***  Growth ratio, Mean±SD  *p* | 3.3±0.7 | 2.0±0.8 | 8.8±1.6  a,b | 6.8±0.8  a,b | 10.7±0.8  a,b,d | 10.8±0.2  a,b,d | 5.4±1.1  b,c,e,f | 5.1±0.2  b,c,e,f | 1.3±0.1  c,d,e,f,g,h |
| ***Prevotella bivia***  Growth ratio, Mean±SD  *p* | 1.4±0.5 | 3.2±1.1 | 6.7±1.5  a,b | 5.2±1.2  a | 0.9±0.1  c,d | 5.9±0.3  a,e | 4.6±1.2  a,e | 7.4±0.9  a,b,e,g | 1.7±0.3  c,d,f, h |

^1^ Statistical differences between bacterial planktonic growths in different culture media were analyzed with one-way ANOVA and Tukey’s multiple comparisons test, *p* < 0.05. **a** Statistical significance when comparing with BHV, **b** when comparing with BHV.Aa, **c** when comparing with NYC, **d** when comparing with NYC.Aa, **e** when comparing with SB, **f** when comparing with SB.Aa, **g** when comparing with sBHI, **h** when comparing with sBHI.Aa.

^2^ The effect of L-ascorbic acid on bacterial growth is presented as fold change relative to the growth in the medium without L-ascorbic acid (fold change = 1, control). This effect was classified as inhibitory (cut-off < 0.75 – fold change), neutral (0.75 ≤ fold change < 1.25), and stimulatory (cut-off ≥ 1.25 – fold change).
