# Supplementary material for: Evaluation of different culture media to support in vitro growth and biofilm formation of bacterial vaginosis-associated anaerobes
Source: PeerJ. 2020 Sep 10;8:e9917. doi: 10.7717/peerj.9917 (PMC7487148; doi:10.7717/peerj.9917)
Supplement: Supplemental Information 4 — 1 Biofilm biomass was quantified using the crystal violet staining method and the resulting solution was assessed by optical density (OD) at 595 nm. 2 Statistical differences between bacterial planktonic growths in different culture media were analyzed with one-way ANOVA and Tukey’s multiple comparisons test, p < 0.05. a Statistical significance when comparing with BHV, b when comparing with BHV.Aa, c when comparing with NYC, d when comparing with NYC.Aa, e when comparing with SB, f when comparing with SB.Aa, g when comparing with sBHI.Aa. [file peerj-08-9917-s004.docx]

**Supplemental Table S2.**

**Biofilm formation of BV-associated bacteria in the nine different culture media over a 72 h period^1^.**

| **Bacteria** | **Culture medium** | | | | | | | | |
| --- | --- | --- | --- | --- | --- | --- | --- | --- | --- |
|  | **BHV** | **BHV.Aa** | **NYC** | **NYC.Aa** | **SB** | **SB.Aa** | **sBHI** | **sBHI.Aa** | **mGTS** |
| ***Gardnerella* sp.**  OD_595nm_, Mean±SD  *p^2^* | 0.2±0.3 | 0.1±0.1 | 1.0±0.1  a,b | 0.7±0.5  b | 1.4±0.1  a,b,d | 1.3±0.1  a,b,d | 1.0±0.1  a,b | 1.1±0.1  a,b | 0.9±0.1  a,b |
| ***Atopobium vaginae***  OD_595nm_, Mean±SD  *p* | 0.1±0.1 | 0.1±0.1 | 1.1±0.1  a,b | 0.1±0.1  c | 0.1±0.1  c | 0.1±0.1  c | 0.1±0.1  c | 0.1±0.1  c | 0.1±0.1  c |
| ***Lactobacillus iners***  OD_595nm_, Mean±SD  *p* | 0.1±0.1 | 0.1±0.1 | 0.7±0.2  a,b | 0.1±0.1  c | 0.2±0.1  c | 0.1±0.1  c | 0.1±0.1  c | 0.1±0.1  c | 0.1±0.1  c |
| ***Mobiluncus curtisii***  OD_595nm_, Mean±SD  *p* | 0.1±0.1 | 0.1±0.1 | 0.6±0.2  a,b | 0.1±0.1  c | 0.1±0.1  c | 0.1±0.1  c | 0.1±0.1  c | 0.1±0.1  c | 0.1±0.1  c |
| ***Peptostreptococcus anaerobius***  OD_595nm_, Mean±SD  *p* | 0.2±0.2 | 0.1±0.1 | 0.9±0.3  a,b | 0.7±0.1  a,b | 1.1±0.2  a,b,d | 0.7±0.1  b,e | 1.1±0.1  a,b | 0.7±0.1  b,e | 0.7±0.1  a,b |
| ***Prevotella bivia***  OD_595nm_, Mean±SD  *p* | 0.5±0.4 | 0.2±0.1 | 1.1±0.1  a,b | 0.3±0.3  c | 1.3±0.4  a,b,d | 1.2±0.1  a,b,d | 0.3±0.1  c,e,f | 1.0±0.3  b | 0.2±0.1  c,e,f,g |

^1^ Biofilm biomass was quantified using the crystal violet staining method and the resulting solution was assessed by optical density (OD) at 595 nm.

^2^ Statistical differences between bacterial planktonic growths in different culture media were analyzed with one-way ANOVA and Tukey’s multiple comparisons test, *p* < 0.05. **a** Statistical significance when comparing with BHV, **b** when comparing with BHV.Aa, **c** when comparing with NYC, **d** when comparing with NYC.Aa, **e** when comparing with SB, **f** when comparing with SB.Aa, **g** when comparing with sBHI.Aa.
